# Thematic Analysis of State Medicaid Buprenorphine Prior Authorization Requirements

**DOI:** 10.1001/jamanetworkopen.2023.18487

**Published:** 2023-06-15

**Authors:** Max Jordan Nguemeni Tiako, Abby Dolan, Matthew Abrams, Kehinde Oyekanmi, Zachary Meisel, Shoshana V. Aronowitz

**Affiliations:** 1 Department of Medicine, Brigham and Women’s Hospital, Boston, Massachusetts; 2 Center for Emergency Care and Policy Research, Perelman School of Medicine at the University of Pennsylvania, Philadelphia; 3 Urban Health Lab, Perelman School of Medicine at the University of Pennsylvania, Philadelphia; 4 Department of Emergency Medicine, Perelman School of Medicine at the University of Pennsylvania, Philadelphia; 5 University of Central Florida, Orlando; 6 Department of Family and Community Health, School of Nursing, University of Pennsylvania, Philadelphia

## Abstract

**Importance:**

Prior authorization (PA) requirements for buprenorphine are associated with lower provision of the medication for the treatment of opioid use disorder (OUD). While Medicare plans have eliminated PA requirements for buprenorphine, many Medicaid plans continue to require them.

**Objective:**

To describe and classify buprenorphine coverage requirements based on thematic analysis of state Medicaid PA forms.

**Design, Setting, and Participants:**

This qualitative study used a thematic analysis of 50 states’ Medicaid PA forms for buprenorphine between November 2020 and March 2021. Forms were obtained from the jurisdiction’s Medicaid websites and assessed for features suggesting barriers to buprenorphine access. A coding tool was developed based on a review of a sample of forms, including fields for behavioral health treatment recommendations or mandates, drug screening requirements, and dosage limitations.

**Main Outcomes and Measures:**

Outcomes included PA requirements for different buprenorphine formulations. Additionally, PA forms were evaluated for various criteria such as behavioral health, drug screenings, dose-related recommendations or mandates or patient education.

**Results:**

Among the total of 50 US states in the analysis, most states’ Medicaid plans required PA for at least 1 formulation of buprenorphine. However, the majority did not require a PA for buprenorphine-naloxone. Four key themes of coverage requirements were identified: restrictive surveillance (eg, requirements for urine drug screenings, random drug screenings, pill counts), behavioral health treatment recommendations or mandates (eg, mandatory counseling or 12-step meeting attendance), interfering with or restricting medical decision-making (eg, maximum daily dosages of 16 mg, requiring additional steps for dosages higher than 16 mg), and patient education (eg, information about adverse effects and interactions with other medications). Eleven states (22%) required urine drug screenings, 6 states (12%) required random urine drug screenings, and 4 states (8%) required pill counts. Fourteen states’ forms (28%) recommended therapy, and 7 (14%) required therapy, counseling, or participation in group sessions. Eighteen states (36%) specified dosage maximums; among them, 11 (22%) required additional steps for a daily dosage higher than 16 mg.

**Conclusion:**

In this qualitative study of state Medicaid PA requirements for buprenorphine, themes were identified that included patient surveillance with drug screenings and pill counts, behavioral health treatment recommendations or mandates, patient education, and dosing guidance. These results suggest that state Medicaid plans’ buprenorphine PA requirements for OUD are in conflict with existing evidence and may negatively affect states’ efforts to address the opioid overdose crisis.

## Key Points

**Question:**

What are US states’ prior authorization (PA) requirements for Medicaid payment for buprenorphine?

**Findings:**

This qualitative study of 50 states’ Medicaid PA forms found that PA requirements for buprenorphine included patient surveillance with drug screenings and pill counts, behavioral health treatment recommendations or mandates, patient education, and dosing guidance.

**Meaning:**

These results suggest that PA form requirements and their associated features may contribute to the undue bureaucratic burdens that curtail access to buprenorphine for opioid use disorder.

## Teaser

This qualitative study describes and classifies buprenorphine coverage requirements based on thematic analysis of Medicaid prior authorization forms from all 50 US states.

## Introduction

Prior authorization (PA) forms are often used to manage access and distribution of costly medications and medications with high safety concerns in the US.^1^ However, some studies suggest that barriers created by the PA process may contribute to delays in treatment and patient withdrawal from care—consequences that outweigh its cost-saving and safety benefits. According to a 2018 survey from the American Medical Association, 92% of physicians reported that the PA process delays patient access to necessary care, and 78% said that it led to patients withdrawing from care.^2^ As insurance coverage remains fragmented across different health care systems and states, rules related to PA vary, and their effects are heterogeneous.^3^

In addition to costs, safety concerns are used to justify the need for prior authorization for certain medications. However, some medications that confer a relatively high morbidity and mortality risk, such as benzodiazepines, require no prior authorizations, while others that are demonstrably safe within a wide dose range are difficult to access in part due to PA requirements. In the context of the US drug overdose crisis, buprenorphine is an example of one such medication. In a qualitative study of physicians who treat patients with opioid use disorder (OUD), many cited PA requirements as a substantial barrier to them prescribing buprenorphine.^4^ To that effect, in 2020, the American Medical Association released a policy brief urging states to remove PA requirements and other barriers to accessing medications for OUD (MOUD) altogether.^5^ Despite these calls to action, obstacles to prescribing MOUD remain. Nationwide rates of overdoses and overdose-related deaths have increased in recent years, and the COVID-19 pandemic has only exacerbated this trend.^6^ In addition to concerns about cost-saving (encouraging the prescription of generic over the brand name, including generic formulations in preferred drug lists) and safety, concerns about diversion also contribute to the impetus to regulate the distribution of buprenorphine more vigorously. However, studies show that nonprescribed consumption of buprenorphine more often stems from barriers to accessing care than recreational use.^7,8^ Furthermore, notwithstanding the motives, more frequent use of nonprescribed buprenorphine is associated with a lower risk of a drug overdose.^9,10^

The progressive removal of PA requirements for MOUD in Medicare plans has allowed researchers to quantify the disadvantages of PA requirements in this domain. A 2021 study found that the removal of PA for MOUD for Medicare Advantage populations led to a decrease in nonprescribed opioid use, an increase in MOUD uptake, and a 19% decrease in the likelihood of relapse for patients who were started on MOUD after removal.^11^ Another study found that PA requirement removal for all Medicare patients was associated with an increase in the administration of buprenorphine and a decrease in health care utilization and expenditures overall.^12^ Regarding state Medicaid PA requirements, a 2019 study found that in states with a PA requirement, addiction treatment programs had half the odds of offering buprenorphine compared with those in states without a PA requirement.^13^ In addition, A 2014 study found that PA requirements for buprenorphine in Massachusetts led to a temporary increase in relapse rates and a reduction in the number of individuals who received greater than 24 mg of buprenorphine daily.^14^ It has since been shown that higher dosages of buprenorphine increase retention in treatment without causing harm.^9,10^

There are net benefits to increasing access to buprenorphine, and experts have argued that there are little to no documented drawbacks to removing buprenorphine PAs.^15,16^ Differences in formulations (mono-product vs buprenorphine-naloxone), mode of administration (eg, sublingual film, tablet, buccal tablets, transdermal patch) and availability of generic options may play a role in states’ PA requirements. For example, the Substance Abuse and Mental Health Services Administration suggests that clinicians reserve buprenorphine mono-products for pregnant patients and/or patients who cannot afford the buprenorphine-naloxone formulation.^17^ Still, several state Medicaid plans continue to uphold PA requirement including for the most commonly used formulation (sublingual buprenorphine-naloxone film) for OUD.^18,19^

All studies focusing on buprenorphine PA requirements across insurance types to date have been quantitative. In this qualitative study, we conducted a thematic analysis of state Medicaid PA forms in order to describe and classify coverage requirements. New knowledge gleaned from examining state Medicaid PA forms may thus inform stakeholders who advocate for the removal of PA requirements for buprenorphine.

## Methods

### Study Type

This qualitative study used a thematic analysis of publicly available health policy documents. Because it is an analysis of publicly available documents that does not involve any patients or patient-related information, this study was deemed exempt from review by the University of Pennsylvania institutional review board and did not require informed consent. This report follows the Standards for Reporting Qualitative Research (SRQR) reporting guideline.^20^

### Data Sources

Medicaid PA forms were extracted through a web search engine and mining of state Medicaid websites from November 2020 to March 2021. We also leveraged existing secondary sources published by the Legal Action Group in 2020 and 2021 as guides in our search.^18,19^

### Data Analysis and Coding

We used the READ (ready materials, extract data, analyze data, and distill findings) approach specific to health policy research described by Dalglish et al.^21^ Three authors (M.J.N.T., A.D., S.V.A.) initially gathered and read a random sample of PA forms in order to identify key themes to expand upon based on form structures, listed requirements, and language used, specifically assessing the forms for features suggesting potential barriers to buprenorphine access (eg, behavioral treatment requirements and random pill count requirements, dosage limitations).

We then developed a coding tool (eAppendix in Supplement 1), which included fields for counseling requirements, urine drug screen requirements, dosage limitations, and other relevant information. Four authors (A.D., S.V.A., M.A., K.O.) then used this tool to code all PA forms independently. The coding team met biweekly throughout the coding process to assess discrepancies or disagreements and reach a consensus. These criteria were then grouped into key themes by 1 author (M.J.N.T.).

## Results

Of the 50 states included in this study, we found that 32 states (64%) required a PA form for coverage of at least 1 formulation of buprenorphine. All 32 of these states required a PA form for the mono-product (buprenorphine-only) formulation. Of these 32, 15 states also required a PA form for the combination product (buprenorphine-naloxone), the formulation most commonly used for OUD. Thirty-nine states (78%) covered generic buprenorphine-naloxone on preferred drug lists (PDL)^22^ without requiring a PA form, whereas 6 states (12%) required PA for genetic buprenorphine-naloxone and did not cover it under their PDLs. Proof of pregnancy was listed as an eligibility criterion for buprenorphine-only formulation on 21 PA forms (42%).

We identified 4 key themes in our analysis: (1) restrictive surveillance, (2) behavioral health treatment, (3) interfering with or restricting medical decision-making, and (4) patient education. Questions specific to each category are listed in the Table, and the eAppendix in Supplement 1 includes all state-specific PA form data tabled by our coding tool.

**Table.** Questions Used to Classify States’ Prior Authorization Forms Into Key Themes

Questions			
Restrictive surveillance	Behavioral health treatment	Interfering with or restricting medical decision-making	Patient education
Does the form ask about checking the PDMP?	Does the form contain questions about therapy?	Are there questions/suggestions about dose reduction or a goal of tapering?	Does the form contain patient education about the medication?
Does the form contain questions about urine drug screens?	If the form contains questions about therapy, are therapy/counseling/groups required?	Are there maximum dosages listed? If so, what are they?	If yes, does it contain information about side effects?
If yes, are urine drug screens required?	Is proof of therapy/counseling/group attendance required?	Are there additional approvals/steps/paperwork needed for a dose higher than 16 mg?	Does it contain information about not mixing with other medications such as benzodiazepines?
Are random urine drug screens required?	Are there a set number of required sessions or a required amount of time spent in therapy/counseling/groups?	Does the clinician need to be or consult with a specialist?	If yes, does it contain information on how the medication works?
Does the form contain questions about pill counts?	Do group sessions need to be “12-step”?	If patient is pregnant, does the clinician need to be a board certified OBGYN?	
If the form mentions pill counts, are pill counts required?	If not required to be 12-step, can the therapy requirement be met with 12-step groups?	Does the form have any additional eligibility criteria for Subutex (buprenorphine-only product)?	
Does the form require random pill counts?			
Are patients banned from seeking care with controlled substances from other clinicians?			
Does the state require a patient contract (even if not a separate document, but just questions on the clinician form) along with the clinician form?			
If yes, does the state provide the contract or just mention that a provider should have one?			
If the state provides one, does it contain intimidating legal language (ie, remind patients that sharing the medication is illegal or even a felony)?			

Abbreviations: OBGYN, obstetrician-gynecologist; PDMP, prescription drug monitoring program.

### Restrictive Surveillance

Eleven states (22%) required urine drug screenings, with 6 states (12%) requiring random screenings. Four states (8%) required pill counts. Additionally, 7 states (14%) required patients to sign a contract outlining regulations to follow in order to receive treatment.

### Behavioral Health Treatment

Fourteen states’ forms (28%) recommended therapy. Seven states’ forms (14%) required proof of therapy, counseling, or participation in group sessions.

### Interfering With or Restricting Medical Decision-making

Eighteen states (36%) specified dosage maximums; among them, 11 (22%) required additional steps for a daily dosage higher than 16 mg. The most common maximum daily dosage listed for buprenorphine or buprenorphine-naloxone was 24 mg per day (Alaska, Idaho, Montana, Pennsylvania, Virginia, and Utah), with no option to increase the daily dosage. Massachusetts required documentation of the medical necessity of dosage beyond 24 mg. A few states had a maximum dosage of 16 mg with no option to raise the dose (Georgia, Kentucky, Oklahoma, Vermont). Some states’ dosage limitations were more nuanced: Wyoming had a maximum daily dosage of 16 mg for the first 2 years of treatment, after which patients would be limited to a maximum dosage of 8 mg per day. Regarding supply with each prescription, only 1 state (Vermont) had a maximum number of days’ supply per prescription fill (14 days).

### Patient Education

Six forms (12%) included patient education about buprenorphine, such as information about side effects, drug-drug interactions, and special considerations such as pregnancy. One state’s PA form (Alabama) makes no mention of pregnancy, while North Dakota’s and Florida’s PA forms require that clinicians ask about pregnancy and breastfeeding. Kentucky’s PA form asks if the patient has been counseled about the risk of neonatal abstinence syndrome and about access to family planning services. In terms of drug-drug interactions, 6 states (12%) included information about the risks of mixing buprenorphine with other medications, such as benzodiazepines.

Lastly, we classified states with PA forms into categories based on the number of items their PA forms contained that corresponded to each of the aforementioned themes. Forms with 0 items relevant to the domain of interest were classified as null. For the patient education and restrictive surveillance themes, we classified forms that had fewer items than the mean as low, and those with more items than the mean were classified as high. The themes behavioral health and interfering with or restricting medical decision-making had a wider spread and greater prevalence across states, therefore we used quartiles, classifying forms below the 25th percentile in number of items as low, those between the 25th and below the 75th percentile as medium, and those at the 75th percentile or above as high.

Five states’ PA forms (10%) ranked high in terms of restrictive surveillance (Alabama, Kentucky, Maine, Montana, and Nebraska). Among them, 3 (6%) ranked high in terms of interfering with or restricting medical decision-making (Montana, Kentucky, and Maine). Six additional states (12%) ranked high in interfering with or restricting medical decision-making (Georgia, Massachusetts, Utah, Pennsylvania, Wyoming, Tennessee). Five states (10%) ranked high in terms of patient education (Alaska, Arkansas, Kentucky, Nebraska, and West Virginia). Among these states, only Kentucky ranked high in interfering with or restricting medical decision-making and restrictive surveillance. The Figure shows a comprehensive classification of each state.

**Figure.**
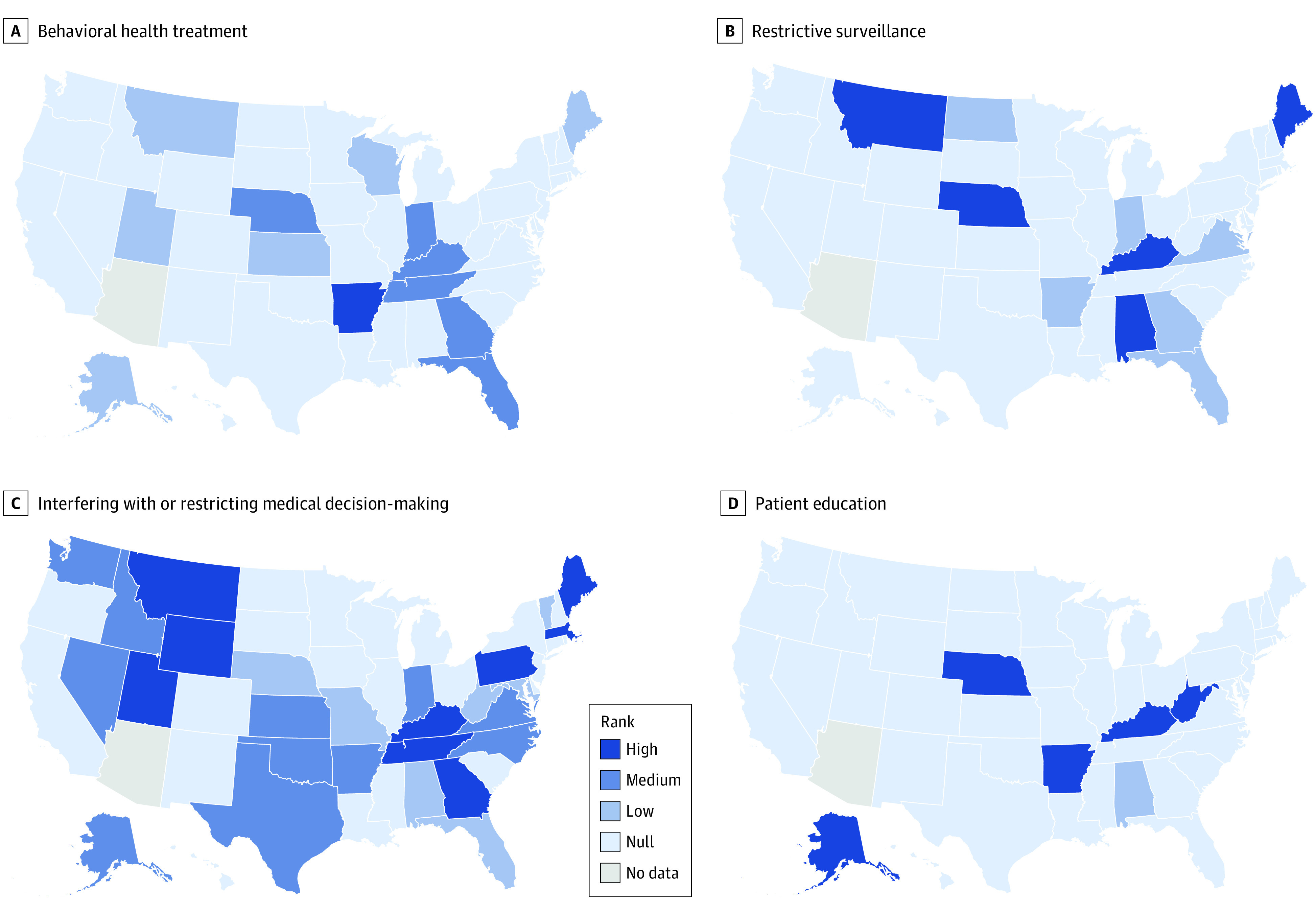
Classification of State Medicaid Prior Authorization Form Requirement Along Key Themes

## Discussion

Our study has 4 main findings. First, most states’ Medicaid plans require PA for at least 1 formulation of buprenorphine, but most do not require a PA for buprenorphine-naloxone, the most common formulation for OUD. Second, a handful of states do not cover even generic formulations of buprenorphine-naloxone under PDLs, thus requiring a PA. Third, PA forms impose a bureaucratic burden on clinicians and patients via restrictive surveillance, interference, and restriction in medical decision-making. Lastly, while patient education is a feature of some PA forms, states with highly restrictive surveillance and interfering with or restricting medical decisions mostly did not include any patient education.

Most states do not require a PA for generic buprenorphine-naloxone, the formulation most commonly used for OUD. However, a few states do not cover generic buprenorphine-naloxone under their PDLs, ruling out cost concerns as a motivation for requiring a PA.^22^ Prior research has shown that lower use of buprenorphine is associated with higher hospitalization and emergency department visit rates, thus increasing costs to payers.^12^ Therefore, while policy makers may cite costs as the driver behind PA requirements, our findings and those of previous studies suggest that in many cases, PA requirements do not lower costs.

Concerns about diversion may also explain restrictions. However, previous research suggests that nonprescribed use of buprenorphine is primarily motivated by lack of access to treatment,^8^ and that people who use nonprescribed buprenorphine do so to treat their withdrawal symptoms. Additionally, more recent evidence shows that any use of buprenorphine contributes to reduced overdose risk, suggesting that while diversion may not be legal, it likely contributes to reducing overall community-level overdose risks where the formal treatment system falls short.

Similarly, restrictions on specific formulations, such as buprenorphine without naloxone (the mono-product), likely stem from concerns about individuals potentially injecting buprenorphine, hence why naloxone was added to buprenorphine as a way to deter misuse.^23^ However, more recent evidence suggests that despite the addition of naloxone to buprenorphine, some people do inject the combined buprenorphine-naloxone formulation,^24,25^ though it is less desirable than the mono-product and may have lower street value.^23^ This evidence calls into question the utility of separating buprenorphine-only from buprenorphine-naloxone administratively by requiring evidence of allergy to naloxone, lack of tolerability, or pregnancy before initiating the mono-product. Ultimately, the ceiling effect of buprenorphine on the mu-opioid receptor, whether administered sublingually or injected intravenously, makes it such that the risk of overdose in adults is negligible, and in fact, higher frequency of nonprescribed buprenorphine use is associated with a lower risk of a drug overdose.^26^ Conversely, while the bioavailability of naloxone in the buprenorphine-naloxone product is reported to be negligible, its adverse effects are not. Some patients report headaches or nausea when taking the combined product, and these symptoms often resolve after switching to the mono-product. Unfortunately, unpleasant adverse effects can lead to patients discontinuing buprenorphine altogether, especially if they face barriers accessing the mono-product.^27^

The administrative burden (time and paperwork completion) on clinicians required by PA forms further illustrates what clinicians have previously reported as barriers to prescribing buprenorphine.^28^ Additionally, aspects of the PA forms that interfere with clinical decision-making, such as dose-specific requirements or maximum daily dosage, are not consistent with existing evidence. Research shows that high-dose buprenorphine is associated with increased retention, especially among patients with more severe OUD.^10^ However, most maximum daily dosages are capped at 16 mg, whereas 24 to 32 mg are generally considered high doses. A 2016 study found that a payer-mandated maximum sublingual buprenorphine dosage of 16 mg per patient was associated with a greater rate of aberrant urine drug tests and lower retention rates compared with patients who received dosages higher than 16 mg. This suggests that currently imposed daily maximums are out of date and may be detrimental to states’ goals of preventing overdoses.^29^

Medicaid insures people with low income, who make up a disproportionate share of people with OUD. The fact that in some states, Medicaid plans still require PA forms while Medicare no longer requires them highlights a great opportunity to further reduce barriers to treatment^30^ and overdose risk among those most affected. Studies show that lifting the PA requirement leads to greater engagement in treatment and improvement in outcomes, yet this evidence has not engendered sufficient change. Furthermore, there is no evidence that PA requirements yield any benefits for patients.

A qualitative study of patients with OUD who self-treated with nonprescribed buprenorphine^31^ found that the demands of formal treatment are a major motivation for opting out, as the one-size-fits-all requirements to engage with mental health treatment may interfere with individuals’ other needs, such as maintaining full-time employment or minimizing travel time due to costs or disabilities. One participant stated: “You have to jump through the hoops with these damn treatment centers. No one wants to go. You can’t work because you have to go to 3 groups a week and, go to case management, see the doctor. […] Treatment centers are set up for failure here. Totally.”^31^ This study also highlights that therapy sessions or meetings could be used as punishment for perceived shortcomings such as tardiness.^31^ This suggests that requirements to attend therapy and other restrictive aspects of PAs deter patients from seeking or remaining in treatment, even though, according to a recent systematic review, no study has found a significant difference in retention between buprenorphine alone and buprenorphine plus behavioral health treatment.^10^ While some patients opt to self-treat, it is likely that a substantial proportion simply opt out of any treatment at all. Though the intent of PA requirements is said to be about reducing costs and ensuring patient safety, states may inadvertently produce death, as evidence shows that removing PA forms ultimately contributes to saving lives.^12,32^

### Limitations

Our study has a few important limitations. We were not able to capture any potential changes in Medicaid-specific buprenorphine PA requirements during the height of the COVID-19 pandemic. Because our study had a qualitative focus, we cannot infer from our findings any effect on patient outcomes, variations in outcome by state based on factors such as population or severity of the overdose epidemic. Still, our findings provide some insights in line with other patient-focused qualitative work on barriers to access to buprenorphine.

## Conclusion

This qualitative study of state Medicaid PA forms for buprenorphine provides insights into how states may increase barriers to access to medications for OUD. The restrictions around different types of buprenorphine formulations and dosages are not in line with the existing evidence. States may be causing undue harm by preserving prior authorization requirements, given evidence of cost-effectiveness and improved patient outcomes when PAs for buprenorphine are lifted. Lifting PA requirements for buprenorphine across all states’ Medicaid plans and reevaluating the tiering of different formulations are important steps toward increasing access to buprenorphine and reducing the adverse effects of the opioid overdose crisis.

## Notes

Supplement 1. **eAppendix.** Initial Coding Tool Developed to Assess Prior Authorization Forms

Supplement 2.
Data Sharing Statement
