# Supplementary material for: Thematic Analysis of State Medicaid Buprenorphine Prior Authorization Requirements
Source: JAMA Netw Open. 2023 Jun 15;6(6):e2318487. doi: 10.1001/jamanetworkopen.2023.18487 (PMC10273019; doi:10.1001/jamanetworkopen.2023.18487)
Supplement: Supplement 1. — eAppendix. Initial Coding Tool Developed to Assess Prior Authorization Forms [file jamanetwopen-e2318487-s001.pdf]

Supplemental Online Content

Nguemeni Tiako MJ, Dolan A, Abrams M, Oyekanmi K, Meisel Z, Aronowitz SV. Thematic analysis of state Medicaid buprenorphine prior authorization requirements. *JAMA Netw Open.* 2023;6(6):e2318487. doi:10.1001/jamanetworkopen.2023.18487

eAppendix. Initial Coding Tool Developed to Assess Prior Authorization Forms

This supplemental material has been provided by the authors to give readers additional information about their work.

© 2023 Nguemeni Tiako MJ et al. *JAMA Network Open.*

eAppendix. Initial Coding Tool Developed to Assess Prior Authorization Forms

1. Does the state require a patient contract (even if not a separate document, but just questions on the clinician form) along with the clinician form?
2. Does the PA form ask about checking the PDMP?
3. Are therapy/counseling/groups required?
3a. Does the form contain language about "patient compliance" with therapy/counseling?
3b. Do groups need to be "12-step"?
3c. Are there a set number of required sessions?
4. Is urine drug screen (UDS) required?
4a. Random or scheduled UDS?
5. Are pill counts required?
5a. Random or scheduled?
6. Are there questions about dose reduction or evaluation if dose is higher than 16mg?
7. Are there additional approvals needed for a dose higher than 16mg?
8. Does the clinician need to be or consult with a specialist?
9. Does the clinician need to be an MD?
10. If patient is pregnant, does the clinician need to be board certified OBGYN?
10a. Does the form use trans-exclusionary language?
11. Does the form have any questions about Subutex (buprenorphine only product)?
11a. Does a non-pregnant patient need to have an allergy to qualify?
**If there is a required patient contract:**
1.Does it contain intimidating legal language? (ie remind patients that sharing the medication is illegal or even a felony)
2.Does it mention prohibition of concurrent substance use?
3. Are patients banned from seeking care with controlled substances from other clinicians?
4. Is there language about the goal being to reduce the dose?
5. Does the form contain patient education about the medication?
6. Must the patient agree to UDS and pill counts?
7. Must the patient agree to pill counts?
8. Does the contract mention lost/stolen medication?
8a. Does it state that lost medication will not be replaced?
9.What language is used to describe buprenorphine? (i.e.: "drugs")
**Any other note-worthy details or text:**

© 2023 Nguemeni Tiako MJ et al. *JAMA Network Open.*
