# Supplementary material for: Thematic Analysis of State Medicaid Buprenorphine Prior Authorization Requirements
Source: JAMA Netw Open. 2023 Jun 15;6(6):e2318487. doi: 10.1001/jamanetworkopen.2023.18487 (PMC10273019; doi:10.1001/jamanetworkopen.2023.18487)
Supplement: Supplement 2. — Data Sharing Statement [file jamanetwopen-e2318487-s002.pdf]

Data Sharing Statement

Nguemeni Tiako. Thematic Analysis of State Medicaid Buprenorphine Prior Authorization Requirements. *JAMA Netw Open*. Published June 15, 2023.
doi:10.1001/jamanetworkopen.2023.18487

Data
Data available: Yes
Data types: Other (please specify)
Additional Information: A repository of Medicaid prior authorization forms collected, and a spreadsheet reporting authors' assessment of each state's form
How to access data: A repository of Medicaid prior authorization forms collected, and a spreadsheet reporting authors' assessment of each state's form
When available: With publication

Supporting Documents
Document types: None

Additional Information
Who can access the data: Anyone requesting the data
Types of analyses: qualitative analyses
Mechanisms of data availability: with a signed data access agreement
Any additional restrictions: n/a
